# Clinical manifestations in patients with PI*MM_Malton_ genotypes. A matter still unsolved in alpha‐1 antitrypsin deficiency

**DOI:** 10.1002/rcr2.528

**Published:** 2020-02-19

**Authors:** Marina Aiello, Alberto Fantin, Chiara Longo, Ilaria Ferrarotti, Giuseppina Bertorelli, Alfredo Chetta

**Affiliations:** ^1^ Department of Medicine and Surgery, Respiratory Disease and Lung Function Unit University of Parma Italy; ^2^ Center for the Diagnosis of Inherited Alpha1‐antitrypsin Deficiency, Department of Internal Medicine and Therapeutics, Pneumology Unit University of Pavia Italy

**Keywords:** Alpha‐1 antitrypsin deficiency, genotype, lung and liver function

## Abstract

We report the genetic variants associated with alpha‐1 antitrypsin deficiency (AATD) in 117 patients admitted to our outpatient clinic and characterized by a serum concentration of AAT lower than 113 mg/dL. We focused on the M‐like heterozygous variant of the *SERPINA1* gene called PI*MM_Malton_, and describe three patients with this variant. While the role of homozygous AATD in liver and pulmonary disease is well established, the association between heterozygous AATD and chronic liver and pulmonary disease is still under investigation. The PI*MM_Malton_ genotype was found in 5.8% of patients with a pathological genotype of AATD and in 14.3% of the subjects when considering only those with intermediate AATD. There were no liver or renal abnormalities in patients with the PI*MM_Malton_ genotype. The PI*MM_Malton_ patients included here showed a normal liver function, and none had renal function abnormalities or abdominal aortic aneurysm. Only a prevalence of lung disease was detected.

## Graphical abstract

The PI*MMMalton patients in our study showed a normal liver function, and none of them had renal function abnormalities or abdominal aortic aneurysm. Only a prevalence of lung disease has been detected.

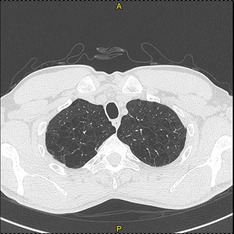

## Introduction

Alpha‐1 antitrypsin deficiency (AATD) is an autosomal co‐dominant genetic disorder characterized by a decreased serum concentration of alpha‐1 antitrypsin (AAT). A deficiency of this protein predisposes to the onset of pulmonary emphysema, bronchial asthma, bronchiectasis, hepatopathy unrelated to other conditions, panniculitis, arterial aneurysms, and vasculitis, such as granulomatosis with polyangiitis 1. AAT functions mainly as a protease inhibitor. A decreased plasma concentration of AAT leads to an imbalance between its protective activity and the proteolytic activity of neutrophil elastase.

AAT, a small glycoprotein encoded by a gene located on chromosome 14, belongs to the serine protease family (Serpin) and is synthesized and released mainly by hepatocytes. The Z variant is the most common, clinically significant, deficient variant, characterized by the replacement of amino acid Glu342 to Lys342. Among other rare pathological variants, the so called “M‐like” variants (e.g. M_Malton_, M_Wurzburg_, M_Procida_, etc.) play an interesting role. These mutations produce an isoelectrophoretic pattern similar to the normal M variants; consequently, a phenotypic analysis can misdiagnose these variants as normal and only the gene sequencing allows their univocal identification 2.

The aim of this study was to analyse the genetic variants associated with AATD in 117 patients admitted to our outpatient clinic and characterized by a serum concentration of AAT lower than 113 mg/dL. Blood samples from these patients were taken in ethylenediaminetetraacetic acid (EDTA) or by using the AlphaKit™ (Whatman, GE Healthcare; Maidstone, UK) test 3. DNA was extracted and amplified through real‐time polymerase chain reaction. The amplification products were then analysed by electrophoresis on 2% agarose gel in TBE buffer (Tris‐Borate‐EDTA) and sequenced through the Sanger enzymatic method. In our study, we focused in particular on the M‐like heterozygous variants of the *SERPINA1* gene called PI*MM_Malton_ (p.F75del, c.227_229del rs775982338). The study was approved by our local Ethics Committee. All patients signed an informed consent form authorizing the use of their data for this study.

Fifty‐two out of the 117 patients undergoing genetic investigation showed a pathological genotype, while 65 patients had a PI*MM genotype. In more detail, the pathological genotypes we found were: PI*MS (24 patients), PI*MZ (16 patients), PI*SZ (three patients), PI*MM_Malton_ (three patients), PI*SS (two patients), PI*MM_Wurzburg_ (two patients), PI*ZZ (one patient), and PI*VZ (one patient). Twenty‐seven out of the 52 patients with a pathological genotype were admitted to our outpatient clinic because of respiratory problems, while the remaining 25 subjects were relatives of the index cases (52% and 48% of the total, respectively).

The patients with the PI*MM_Malton_ variant corresponded to 6% of all the patients analysed. A discussion of these three clinical cases will follow below.

## Case Series

### Case 1

A 43‐year‐old male subject, Italian Caucasian, affected by asthma and atopy, with prick tests positive since the childhood for dog and cat dander and grass pollen, treated with inhaled corticosteroids, long‐acting ß_2_‐agonists (ICS/LABAs) and long‐acting muscarinic antagonists (LAMAs). The patient referred to our outpatient clinic for recurrent bronchitis and exertional dyspnoea. He was a former smoker (about 10 cigarettes a day for 20 years, 10 pack/years) at the time of the visit and a manual worker with no previous exposure to any toxic or irritating agent.

Upon auscultation, he presented a slightly reduced vesicular murmur, with wheezing during forced expiration; oxygen saturation (SatO_2_) was 96% breathing room air and the heart rate was 71 bpm. Arterial blood gas measurement in breathing room air produced the following results: pH 7.46, pCO_2_ 37 mmHg, pO_2_ 91 mmHg, HCO_3_ 27 mmoL/L, and sO_2_ 97%.

AAT serum concentration was 64 mg/dL, with a PI*M2M_Malton_ genotype.

Complete abdomen ultrasound exam did not identify hepatic or aneurysmal disease. Total and fractioned bilirubin, creatine phosphokinase (CPK), gamma‐glutamyl transferase (gamma GT), cholinesterase, aspartate aminotransferase (AST), and alanine aminotransferase (ALT) blood tests were in the normal range, as well as urea and creatinine; anti‐neutrophil cytoplasmic antibodies (C‐ANCA) and anti‐myeloperoxidase antibodies (anti‐MPO) were negative, while a marked increase in total IgE was detected (1260 IU/mL). Autoantibodies and eosinophils were also in the normal range.

**Figure 1:**
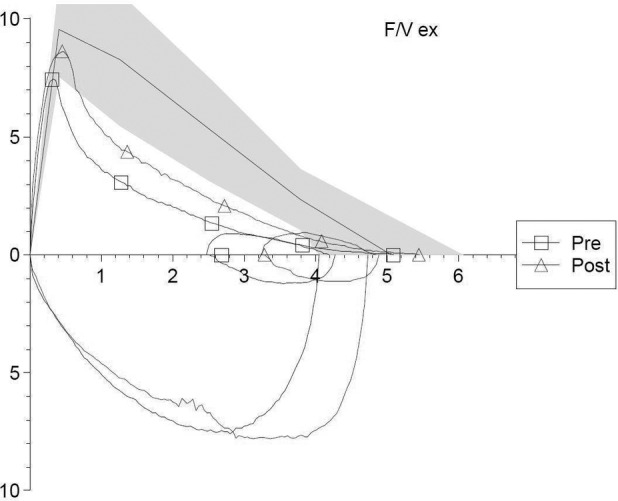
Flow‐volume loop of the Case 1 patient*,* before (“pre”) and after (“post”) the administration of 400 μg of salbutamol.

Lung function tests showed an extremely severe obstructive ventilatory defect. The bronchial reversibility test was positive (Fig. 1; Table 1). The chest high resolution computed tomography (HRCT) excluded pulmonary emphysema, bronchiectasis and fibrosis. The six‐minute walking test (6MWT), performed without oxygen supplementation, did not register any clinically significant desaturation but a slight reduction in the distance walked (440 m measured/546 m predicted). Echocardiographic parameters were in the normal range.

**Table 1:** Pre‐ and post‐bronchodilator spirometric values for patients corresponding to Cases 1 and 3.

Variables	Case 1 pre	Case 1 post	Change, %	Case 3 pre	Case 3 post	Change, %
FEV_1_, L	1.88	2.55	36.0	3.14	3.30	5
FEV_1_, % predicted	46.0	63.0		85.0	89.0	
FVC, L	4.71	4.98	6.0	4.92	4.86	−1
FVC, % predicted	95.0	101.0		108.0	107.0	
FEV_1_/FVC, %	39.9	51.2	28.0	63.8	67.9	7
TLC, L	8.77			7.18		
TLC, % predicted	121.0			104.0		
RV, L	3.81			2.08		
RV, % predicted	193.0			100.0		
RV/TLC, %	43.4			28.9		
DLCO, % predicted	89.0			67.0		
KCO, % predicted	96.0			69.0		

FEV_1_, forced expiratory volume in one second; FVC, forced vital capacity; FEV_1_/FVC, forced expiratory volume in one second to forced vital capacity ratio; TLC, total lung capacity; RV**,** residual volume; RV /TLC, residual volume to total lung capacity ratio; DLCO, diffusing capacity for carbon monoxide; KCO, transfer coefficient of the lung for carbon monoxide.

### Case 2

A 62‐year‐old male subject, Italian Caucasian, in good health conditions. The diagnosis of AATD was made following genotype analysis performed on the son of the subject (the patient in Case 1, with a PI*M2M_Malton_ genotype). The patient had an AAT serum concentration of 69 mg/dL, with a PI*M3M_Malton_ genotype. He was a former smoker (about four cigarettes a day for 5 years, 1 pack/years), and worked as a sandblaster craftsman, reporting powder working exposure.

The patient did not take any drug, and did not complain of any respiratory symptoms. The 6MWT, performed without oxygen supplementation, did not register a clinically significant desaturation and the distance walked was normal (424 m measured/530 m predicted). SatO_2_ was 100% breathing room air and the heart rate was 66 bpm. Upon thoracic auscultation, the vesicular murmur was preserved, with no pathological sound. Lung function tests were in the normal range (Table 2). Blood count, liver function tests and renal function were in the standard reference range, while the total IgE concentration was 429 IU/mL. Chest X‐ray showed no abnormality. Liver and abdominal ultrasound exams were negative for liver or aneurysmal disease. Prick tests were positive for dust mites, whereas bronchial hyperactivity assessed through the methacholine (MCh) test was negative.

**Table 2:** Lung function tests of Case 2 patient.

Variables	Case 2
FEV_1_, L	3.46
FEV_1_, % predicted	108.2
FVC, L	4.34
FVC, % predicted	107.7
FEV_1_/FVC, %	79.6
TLC, L	7.14
TLC, % predicted	107.1
RV, L	2.36
RV, % predicted	101.8
RV/TLC, %	33.1

FEV_1_, forced expiratory volume in one second; FVC, forced vital capacity; FEV_1_ / FVC, forced expiratory volume in one second to forced vital capacity ratio; TLC, total lung capacity; RV**,** residual volume; RV /TLC, residual volume to total lung capacity ratio.

### Case 3

A 50‐year‐old male subject of Tunisian origin, followed by our outpatient chronic obstructive pulmonary disease (COPD) clinic, a former smoker (about 10 cigarettes a day for 30 years, 15 pack/years) treated with a LAMA‐based inhalation therapy, and with previous monolateral spontaneous pneumothorax, treated with thoracic drainage. He worked as a driver. The patient presented digital hippocratism and, upon chest auscultation, the vesicular murmur was greatly reduced.

**Figure 2:**
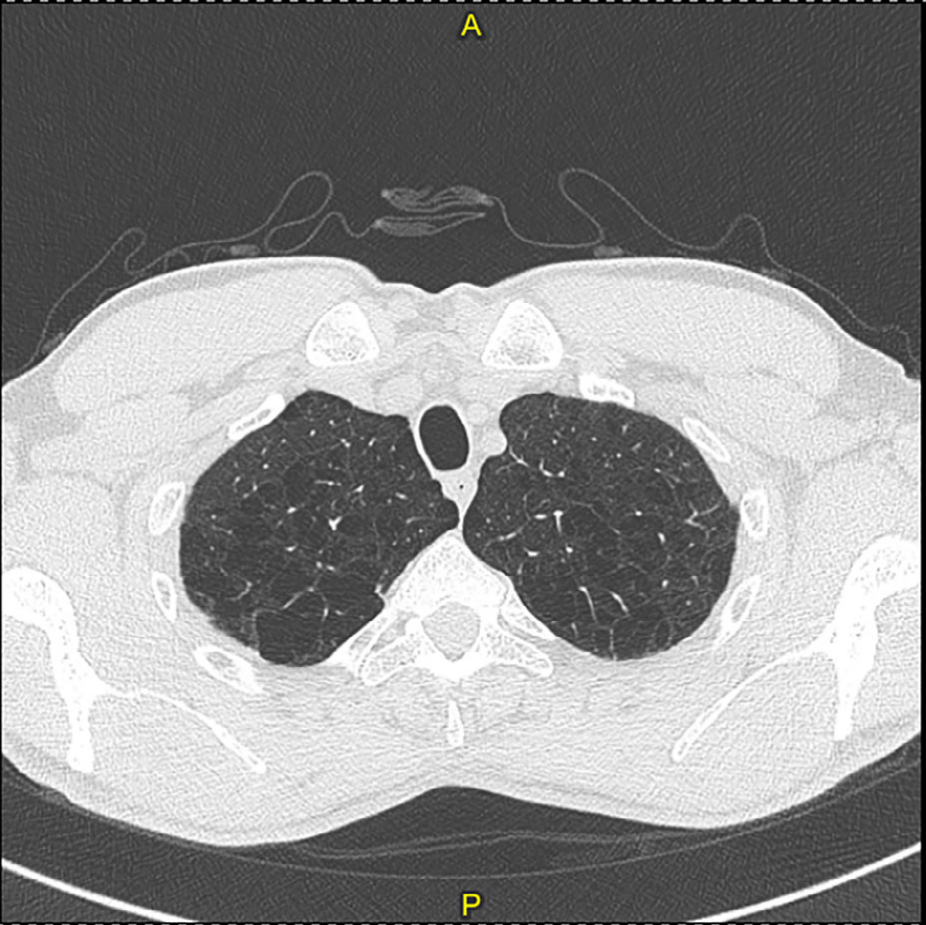
High resolution computed tomography of the Case 3 patient, showing upper lobe panlobular emphysema.

Arterial blood gas analysis showed values in the normal range considering the subject's age: pH 7.35, pCO_2_ 42.9 mmHg, pO_2_ 89.5 mmHg, HCO_3_ 22.6 mmol/L, and sO_2_ 97%. The 6MWT, performed without oxygen supplementation, did not register any clinically significant desaturation but a slight reduction in the distance walked (411 m measured/512 m predicted). Electrocardiogram (ECG) parameters were within the normal limits. The chest HRCT showed a severe diffuse centrolobular and paraseptal emphysema (Fig. 2).

**Figure 3:**
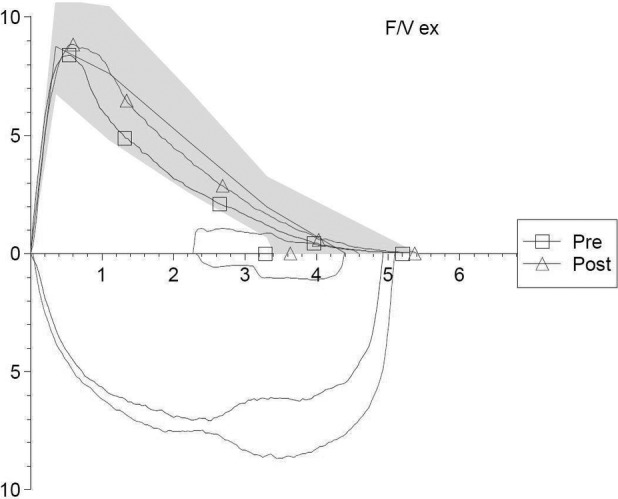
Flow‐volume loop of the Case 3 patient*,* before (“pre”) and after (“post”) the administration of 400 μg of salbutamol.

Pulmonary function tests revealed a mild airway obstruction, with a negative bronchial reversibility test (Fig. 3; Table 1).

Since the AAT serum concentration was lower than normal (87 mg/dL), the patient underwent a genetic investigation with verification of the PI*M1M_Malton_ genotype. Blood chemistry tests (total and fractionated bilirubin, CPK, gamma GT, cholinesterase, AST, ALT and renal function indices) were in the normal range. Liver and abdominal ultrasound exams were negative for liver or aneurysmal disease.

## Discussion

Our study revealed the frequency of rare genetic variants in a wide cohort of subjects affected by AATD, where the patients with PI*MM_Malton_ and PI*MM_Wurzburg_ genotypes were respectively 5.8% and 3.8% of all patients with a pathological genotype. However, when only the subjects with intermediate AATD were considered, the prevalence of these two genotypes increased to 14.3% and 9.5%, respectively. These frequencies are close to the frequency of the PI*MM_Malton_ genotype among patients with intermediate AATD included in the Italian National Registry (9.3%) 4. The Alpha‐1 Foundation Research Registry Network 5 includes subjects with intermediate AATD showing a prevalence of 5.3% of rare AAT variants, lower than in the Italian National Registry.

Regarding the M_Malton_ variant, Cox et al. 6 found the M_Malton_ genotype in a family without hepatic or pulmonary disease. However, the study was limited by the fact that all the patients were under 30 years of age, and therefore could not have had the time to develop the aforementioned pathologies.

Sproule et al. found a severe emphysema in four patients with PI*type M_Malton_Z, however they did not find any difference in lung function tests between heterozygotes with PI type MM_Malton_ and subjects with PI*MM. The authors showed a synergistic effect of cigarette smoke and intermediate AATD, in fact the PI*MM_Malton_ genotypes were associated with a significantly impaired lung function in smokers only 7.

Other authors, while confirming the association between PI*M_Malton_Z and a high risk of developing emphysema, in contrast to the study by Sproules et al.*,* did not find any evidence suggesting that smokers with PI*MM_Malton_ genotypes and intermediate AAT serum concentration have a greater risk of developing emphysema than smokers with normal AAT serum concentration 8.

Reid and co‐workers presented a patient homozygous for the variant PI*M_Malton_ and affected by severe AATD who developed emphysema, cirrhosis and hepatocellular carcinoma 9.

Joly et al. evaluated four patients with M_Malton_ type M‐like genotype, and in particular a patient with PI*MM_Malton_/MM_Malton_ genotype, two PI*MM_Malton_/Z patients and a PI*M/M_Malton_ patient. In the two patients with double heterozygosis, the clinical picture was represented by COPD, without hepatic alterations; the homozygous patient, on the other hand, was affected by hepatic cirrhosis, with small bronchiectasis but without emphysema; the last case, with a simple heterozygous genotype, suffered from end‐stage renal failure, but without liver or lung abnormalities 10.

Figueira et al. have recently shown that patients with intermediate AATD and PI*MM_Malton_ genotype have a high incidence of lung disease without relevant hepatic manifestations 11.

Consistently with the Figueira et al. findings, the patients with a PI*MM_Malton_ genotype did not show any liver or renal abnormalities, but only pulmonary pathologies, that is atopy and asthma in Case 1, atopy in Case 2, emphysema and pneumothorax in Case 3. In addition, two out of the three patients had a moderate‐to‐severe lung function impairment.

In Case 2, the PI*MM_Malton_ genetic defect was associated with good healthy conditions and a normal pulmonary, hepatic, and renal clinical picture. This aspect highlights the fact that, although the patients described in Cases 1 and 2 are relatives, have the same genetic defect and the same exposure to cigarette smoking, the AATD can be clinically expressed in completely different ways. This observation underscores how the presence of other non‐environmental factors, such as genetic modifiers not yet identified, can play an important role in the evolution of the clinical manifestations in these patients 12.

In addition to normal liver function, none of the three patients had abdominal aortic aneurysms. The three patients included in our study were ex‐smokers. It has been reported in literature that smoking, in addition to potentiating lung injury, reduces the antiprotease activity of the AAT molecule by approximately 2000 times 13, making it an important, avoidable factor for the development of emphysema. As for smoking, it is interesting to note that, among patients with pulmonary disease, more than 50% of subjects are ex‐smokers. Smoking cessation is therefore a very important intervention to be implemented 14.

In conclusion, our data showed that the rare M‐like variants with a PI*MM_Malton_ genotype represent a high percentage among patients with intermediate AATD, that appear to be mostly affected at respiratory level, with a strong association to cigarette smoke exposure, without an evident hepatic involvement.

### Disclosure statements

Appropriate written informed consent was obtained for publication of this case series and accompanying images.
